# Exploring Collaboration Enjoyment and Decisional Uncertainty: Actor-Partner Effects in Advanced Care Planning

**DOI:** 10.1093/geroni/igab046.2785

**Published:** 2021-12-17

**Authors:** Shirin Hiatt, Jia-Wen Guo, Lee Ellington, Djin Tay

**Affiliations:** 1 Oregon Health & Science University, Portland, Oregon, United States; 2 University of Utah, Salt Lake City, Utah, United States

## Abstract

Caregivers are often engaged in decision making with and for patients. However, the role of patient-caregiver interpersonal processes on decisions about advance care planning (ACP) are not well known. This secondary data analysis examined the effects of patient-caregiver enjoyment about collaboration regarding choices for life-sustaining treatment on patients’ and caregivers’ decisional uncertainty following a dyadic ACP intervention. A purposive sample of 18 adult home health patients and their informal caregivers (N=36) participated in a one-group pretest posttest study. The Interpersonal Enjoyment subscale of the Perceptions of Collaboration Questionnaire and the Decisional Uncertainty subscale of the Decisional Conflict Scale were administered using parallel questionnaires. The Actor-Partner Interdependence Model (APIM) was used to examine actor and partner effects of patients and caregivers’ interpersonal enjoyment on their uncertainty in decisions about ACP before and after the intervention. The mean age was 68.2±9.6 years for patients and 61.3±13.6 years for caregivers. The majority of patients (61.1%) and caregivers (72.2%) were female and married (55.6% and 66.7%, respectively). Almost all were non-Hispanic White (97.0%). Patients’ and caregivers’ interpersonal enjoyment and uncertainty scores were similar before the intervention. A significant partner effect between greater interpersonal enjoyment among caregivers at pretest and greater patient uncertainty at posttest (β=0.44, p=.037) was found. Previous analyses found that overall patients improved in decisional uncertainty at posttest. However, these findings suggest that for some dyads, interpersonal factors can negatively affect patients’ decisional certainty. Future research is needed to verify this finding with a larger sample.

